# Predicting drug polypharmacology using a novel surface property similarity-based approach

**DOI:** 10.1186/1758-2946-3-S1-O19

**Published:** 2011-04-19

**Authors:** Violeta I Perez-Nueno, Vishwesh Venkatraman, Lazaros Mavridis, David W Ritchie

**Affiliations:** 1 INRIA Nancy – Grand Est, LORIA, 615 rue du Jardin Botanique, 54506 Vandoeuvre-lès-Nancy, France

## 

In recent years, polypharmacology is becoming an increasingly important aspect in drug design. For example, pharmaceutical companies are discovering more and more cases in which multiple drugs bind to a given target (promiscuous targets) and in which a given drug binds to more than one target (promiscuous ligands). Both of these phenomena are clearly of great importance when considering drug side-effects. Given that screening drugs against all the proteins expressed by the human genome is infeasible, several computational techniques for predicting the pharmacological profiles of drugs have been developed, ranging from statistical analyses of chemical fingerprints and biological activities [1] to 3D docking of ligand structures into protein pockets.

Here we present a novel shape-based approach which uses spherical harmonic (SH) representations [2,3] to compare molecular surfaces and key surface properties very efficiently. This approach compares targets by the SH similarity of their ligands and also of their binding pockets. This allows promiscuous ligands and targets to be identified and characterized.

In this contribution, we present details of our approach applied to a subset of the MDL Drug Data Report (MDDR) database containing 65367 compounds distributed over 249 diverse pharmacological targets for which experimental binding information is known. The similarity of each ligand to each target’s ligand set is quantified and used to predict promiscuity. To our knowledge, this is the largest all-against-all polypharmacological study to have been carried out using shape-based techniques. We compare our promiscuity predictions with computational and experimental results obtained by Keiser *et al.*[4]. We also analyse the correlation between binding pocket shapes and ligand-based promiscuity predictions using the ligand and pocket shape similarity matrices.
